# Subacute cerebellar ataxia following respiratory symptoms of COVID-19: a case report

**DOI:** 10.1186/s12879-021-05987-y

**Published:** 2021-03-24

**Authors:** Jana Werner, Ina Reichen, Michael Huber, Irene A. Abela, Michael Weller, Ilijas Jelcic

**Affiliations:** 1 Department of Neurology, University Hospital Zurich, Zurich, Switzerland; 2 Institute of Medical Virology, University of Zurich, Zurich, Switzerland; 3 Department of Infectious Diseases and Hospital Epidemiology, University Hospital Zurich, Zurich, Switzerland; 4 Neuroimmunology and Multiple Sclerosis Research Section, Department of Neurology, University Hospital Zurich, Zurich, Switzerland

**Keywords:** Post-infectious neurological disease, Post-infectious cerebellar ataxia, SARS-CoV-2 infection, COVID-19, Case report

## Abstract

**Background:**

Severe acute respiratory syndrome virus 2 (SARS-CoV-2) is spreading globally and causes most frequently fever and respiratory symptoms, i.e. Coronavirus disease 2019 (COVID-19), however, distinct neurological syndromes associated with SARS-CoV-2 infection have been described. Among SARS-CoV-2-infections-associated neurological symptoms fatigue, headache, dizziness, impaired consciousness and anosmia/ageusia are most frequent, but less frequent neurological deficits such as seizures, Guillain-Barré syndrome or ataxia may also occur.

**Case presentation:**

Herein we present a case of a 62-year-old man who developed a subacute cerebellar syndrome with limb-, truncal- and gait ataxia and scanning speech 1 day after clinical resolution of symptomatic SARS-CoV-2 infection of the upper airways. Apart from ataxia, there were no signs indicative of opsoclonus myoclonus ataxia syndrome or Miller Fisher syndrome. Cerebral magnetic resonance imaging showed mild cerebellar atrophy. SARS-CoV-2 infection of the cerebellum was excluded by normal cerebrospinal fluid cell counts and, most importantly, absence of SARS-CoV-2 RNA or intrathecal SARS-CoV-2-specific antibody production. Other causes of ataxia such as other viral infections, other autoimmune and/or paraneoplastic diseases or intoxication were ruled out. The neurological deficits improved rapidly after high-dose methylprednisolone therapy.

**Conclusions:**

The laboratory and clinical findings as well as the marked improvement after high-dose methylprednisolone therapy suggest a post-infectious, immune-mediated cause of ataxia. This report should make clinicians aware to consider SARS-CoV-2 infection as a potential cause of post-infectious neurological deficits with an atypical clinical presentation and to consider high-dose corticosteroid treatment in case that a post-infectious immune-mediated mechanism is assumed.

## Background / introduction

Severe Acute Respiratory Syndrome Coronavirus-2 (SARS-CoV-2) is currently spreading worldwide causing coronavirus disease 2019 (COVID-19) pandemic. COVID-19 is characterized by a variety of symptoms ranging from mild fever and cough to SARS and multi-organ dysfunctions with substantial mortality in severe cases [1]. COVID-19-associated neurological disorders described so far include (meningo-)encephalitis, hypoxic encephalopathy, acute cerebrovascular disease, acute demyelinating encephalomyelitis, acute hemorrhagic necrotizing encephalopathy and Guillain-Barré syndrome [2–4]. Among the most common neurological symptoms were fatigue, headache, dizziness, impaired consciousness, anosmia/ageusia and vomiting [2, 3]. Other neurological symptoms in the context of COVID-19 have been less commonly described, e.g. seizures in < 2% of patients and ataxia in < 1% of patients [2, 3]. Cases with ataxia included acute cerebellar ataxia (2 cases during COVID-19, 1 case without typical COVID-19 symptoms) [5–7], acute cerebellar ataxia and myoclonus (1 case during COVID-19 and 8 cases after COVID-19) [8–13], opsoclonus myoclonus ataxia syndrome (2 cases during COVID-19 and 6 cases after COVID-19) [12–17], ataxia caused by Miller Fisher syndrome (8 cases during COVID-19 and 1 cases after COVID-19) [18–21] and ataxia associated with encephalopathy (8 cases during COVID-19) [22–26]. Here, we describe a case of a subacute cerebellar ataxia without myoclonus, opsoclonus, oculomotor deficits, sensory deficits or areflexia after recovery from SARS-CoV-2 infection of the upper airways.

## Case presentation

A 62-year old male patient was referred to our center with subacute onset of a cerebellar syndrome manifesting in writing disability and severe gait instability. Sixteen days earlier, he had developed acute-onset cough and high fever for 7 days, and 4 days after onset of upper airway symptoms, he experienced acute ageusia and anosmia for eleven days. Medical history was unremarkable. Clinical, imaging and laboratory findings are summarized in Table 1.

**Table 1:** Symptoms and therapy (a), imaging findings (b), laboratory findings (c) and CSF characteristics (d)

**a) Symptoms and therapy**			
Respiratory symptoms	day 1 – day 8		
Anosmia and ageusia	day 4 – day 15		
Onset of ataxia	day 16		
Scale for the assessment and rating of ataxia (SARA)			
•day 16	14 points		
•day 35	5 points		
•day 119	1 point		
Therapy with acyclovir 3 × 840 mg/d iv	day 29 – day 34		
Therapy with methylprednisolone 500 mg/d iv	day 29 – day 33		
**b) Imaging**			
Brain-MRI	Slight generalized brain atrophy with accentuation of atrophy in the cerebellum		
FDG-PET	No signs of metabolically active malignancies, no cerebellar hyper- or hypometabolism		
Chest X-Ray and -CT scan	Bipulmonary opacities accentuated in both upper lobes (inflammatory infiltrates)		
**c) Laboratory findings**	**Result**		**Normal range**
Nasopharyngeal swab for SARS-CoV-2 RNA	positive on day 18		negative
	negative on day 22		
Creatin kinase (U/L)	38		< 190
Leukocyte count (10^9^/L)	7.84		3.0–9.6
Thrombocyte count (10^9^/L)	595 10^9^/L		143–400
Hemoglobin (g/L)	150		134–170
C-reactive protein (mg/L)	3.9		< 5
Anti-neuronal antibodies*	negative		negative
**d) CSF characteristics**			
	**Day 23**	**Day 33**	
CSF Cell Number (/ul)	< 1	4	≤4
CSF Lactate (mmol/L)	1.5	2.3	1.7–2.6
Glucose Ratio (CSF/Serum)	0.7	0.6	> 0.5
Albumin Ratio (CSF/Serum)	6.4 × 10^3^		< 8.1 × 10^3^
Virus PCRs in CSF**	negative	not done	negative
Metagenomic virus sequencing	not done	negative	negative
Oligoclonal bands (OCB)***	type 4 (identical OCB in CSF and serum)	type 4 (identical OCB in CSF and serum)	type 1 (no OCB in serum and CSF)
Intrathecal synthesis of IgG (i.e. CSF/serum antibody index) reactive against			
•SARS-CoV-2 NP****	inconclusive	0.64	< 1.5
•SARS-CoV-2 S1	inconclusive	0.99	
•SARS-CoV-2 S2	inconclusive	0.19	
•Herpes simplex virus type 1/2	inconclusive	negative	< 1.5
•Varicella zoster virus	inconclusive	1.01	< 1.5
•Borrelia burgdorferi	negative	not done	< 1.5

* anti-neuronal antibodies: anti-CASPR2, −LGI1, −Glut, −GlyR, −Hu, −Ri, −Yo, −Amphiphysin, −CV2 (CRMP5), −Ta/Ma2, −Ma1, −SOX1, −GAD65, −ZIC4, −Tr antibodies in CSF and serum; and anti-MOG in serum

** Viral pathogens: PCR for herpes simplex virus type 1 (HSV-1), cytomegalovirus (CMV), JC polyomavirus (JCV), SARS-CoV-2, varicella zoster virus (VZV);

*** *OCB type 1* no oligoclonal bands in CSF and serum, i.e. no intrathecal IgG production, *OCB type 2* oligoclonal IgG bands only in CSF, i.e. intrathecal IgG production, *OCB type 3* oligoclonal bands in CSF and serum with additional bands in CSF, i.e. intrathecal IgG production, *OCB type 4* identical oligoclonal bands in CSF and serum, i.e. no intrathecal IgG production, *OCB type 5* monoclonal IgG bands in CSF and serum

**** CSF/serum-antibody index was calculated according to Reiber [14] and CSF/serum-antibody index > 1.5 indicated intrathecal antigen-specific antibody production

**Fig. 1:**
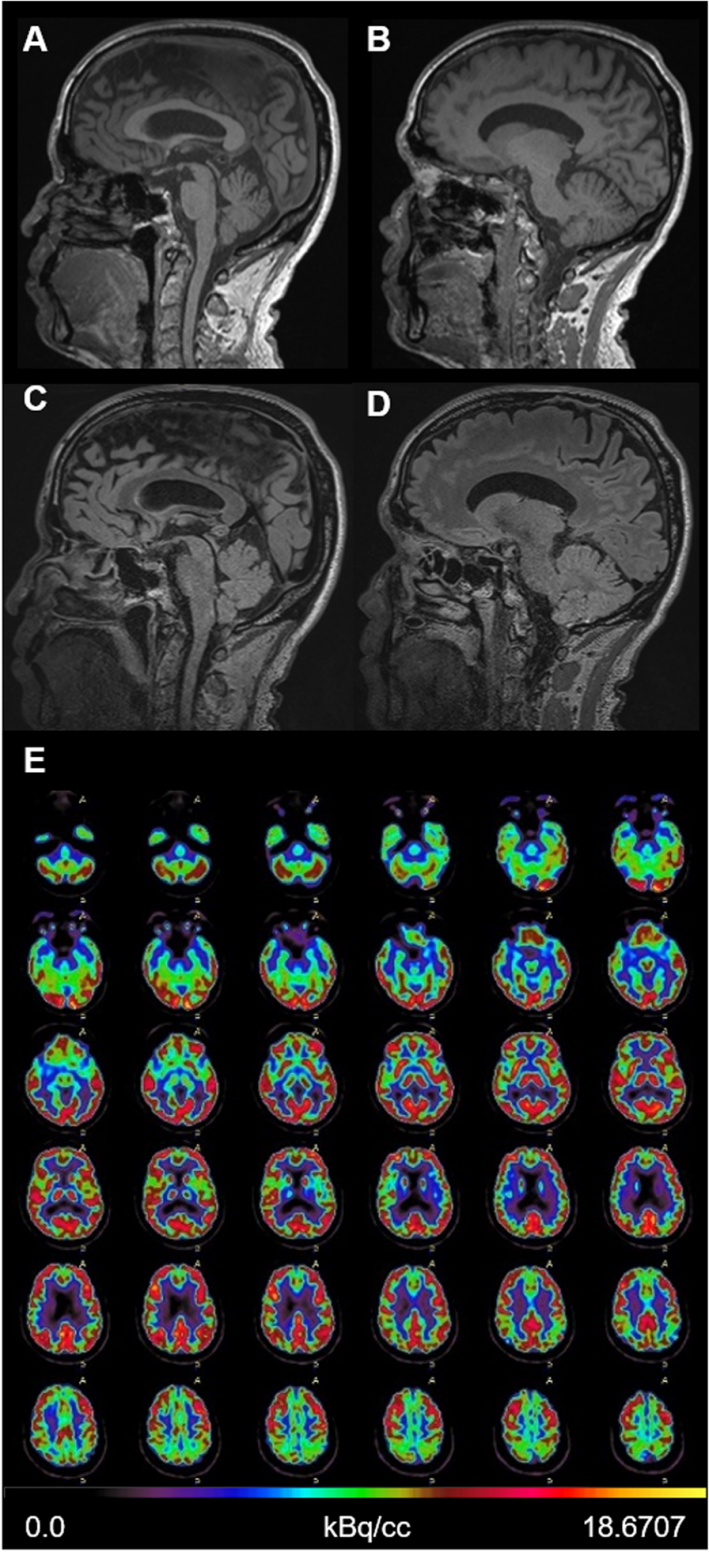
**a and b:** Sagittal T1-weighted magnetic resonance imaging sequences 5 days after onset of ataxia, i.e. 21 days after onset of COVID-19-related respiratory symptoms. **c and d:** Sagittal T2–fluid attenuated inversion recovery magnetic resonance imaging sequences 5 days after onset of ataxia, i.e. 21 days after onset of COVID-19-related respiratory symptoms. **e:** Brain FDG-positron-emission tomography/computed tomography imaging 13 days after onset of ataxia, i.e. 29 days after onset of COVID-19-related respiratory symptoms

The patient presented with a cerebellar syndrome with slightly scanning speech and limb-, truncal- and gait ataxia at admission, i.e. 16 days after onset of COVID-19 respiratory illness and 1 day after resolution of symptoms of COVID-19 affecting the upper respiratory tract. Examination of the eye movements including vertical and horizontal saccades, saccadic pursuit and vestibulo-ocular reflex, was normal. No pathological nystagmus was observed. No opsoclonus or myoclonus was observed. Sensory functions including vibration sense, joint position sense, light touch, pin prick and temperature sensation, were normal; muscle tone, shape, strength and deep tendon reflexes were also normal. His vital signs on admission were normal. Lung auscultation was regular, but CT of the chest showed bipulmonary opacities. He tested positive for SARS-CoV-2 RNA via reverse-transcription polymerase chain reaction (RT-PCR) of nasopharyngeal swab using the Roche Cobas SARS-CoV-2 test (Roche, Basel, Switzerland) on day 2, but tested negative again on day 6 of hospital admission. Cerebral MRI imaging (Fig. 1a-d) showed a mild cerebellar atrophy of unclear etiology, in particular, the patient did not have a history or signs of pre-existing cerebellar disease such as alcohol abuse or gluten hypersensitivity. We ruled out paraneoplasia by whole-body FDG-PET and screening for anti-neuronal antibodies including anti-CASPR2, −LGI1, −Glut, −GlyR, −Hu, −Ri, −Yo, −Amphiphysin, −CV2 (CRMP5), −Ta/Ma2, −Ma1, −SOX1, −GAD65, −ZIC4, −Tr antibodies in cerebrospinal fluid (CSF) and serum, and anti-MOG antibodies in serum. FDG-PET was performed 13 days after onset of cerebellar symptoms and showed normal cerebellar and cerebral metabolism (Fig. 1e). Laboratory results showed no increase in inflammatory markers in peripheral blood, in particular CRP and white blood cell count were normal. CSF testing including cell count, protein, CSF/serum albumin ratio and Reibergram was normal. Isoelectric focusing revealed identical oligoclonal bands in CSF and serum (type 4, see Table 1), indicating systemic inflammation of various etiology but no intrathecal IgG production. Notably, SARS-CoV-2 RT-PCR [27] from CSF was negative. In addition, no viral nucleic acid was detected in metagenomic virus sequencing [28] from CSF. An in-house developed, bead-based antibody assay (ABCORA, Luminex technology) for detection of IgG antibodies against subunit 1 (S1) and subunit 2 (S2) of the spike protein and nucleoprotein (NP) of SARS-CoV-2 was used to screen for SARS-CoV-2-specific antibodies in serum and CSF. We calculated CSF/serum-antibody index according to Reiber [29], and CSF/serum-antibody index > 1.5 indicated intrathecal antigen-specific antibody production. SARS-CoV-2-specific antibodies were positive in serum, but there was no intrathecal SARS-CoV-2-specific antibody production. PCR testing of CSF for DNA of herpes simplex virus type 1 (HSV-1), cytomegalovirus (CMV), JC polyomavirus (JCV) and varicella zoster virus (VZV) were also negative. No intrathecal antibody production reactive to HSV-1, HSV-2, VZV or *Borrelia burgdorferi* was detectable.

Empirical treatment with systemic acyclovir 3x10mg/kg body weight/day was given over 6 days, as herpes viral cerebellitis could not be excluded until day 33. The possibility of cerebellar infection with SARS-CoV-2 was discarded because of the normal CSF cell count and the absence of SARS-CoV-2 RNA and intrathecal SARS-CoV-2-specific antibody production. Other causes such as infections with other viruses, other autoimmune and/or paraneoplastic diseases or intoxication were excluded by details of the past medical history, results of the CSF analysis, auto-antibody screening and FDG-PET. In order to treat a possible post-infectious etiology, namely a post-infectious cerebellar ataxia, we additionally started high-dose methylprednisolone 500 mg/day i.v. over 5 days without subsequent tapering. The cerebellar syndrome improved slowly and slightly over a course of 20 days before acyclovir and corticosteroid treatment, and improved more rapidly with the steroid treatment, which was initiated 14 days after resolution of COVID-19-related respiratory symptoms, i.e. 13 days after onset of ataxia and 7 days after negative SARS-CoV-2 nasopharyngeal swab. The Scale for the Assessment and Rating of Ataxia (SARA ataxia score) improved from SARA 14 points on admission (onset of ataxia, i.e. day 16 after onset of COVID-19) to SARA 5 points 6 days after beginning of steroid treatment (i.e. day 35 after onset of COVID-19), and SARA 1 point nearly 4 months after beginning of steroid treatment (i.e. day 119 after onset of COVID-19). Steroid treatment was well tolerated without re-occurrence of COVID-19 symptoms.

## Discussion and conclusions

We strongly assume a subacute post-infectious cerebellar ataxia with near-complete remission to be the cause of the neurological deficits which occurred after resolution of SARS-CoV-2-induced respiratory illness [30]. We cannot exclude that ataxia occurred by pure chance, but the association in time suggests a link between COVID-19 and subsequent ataxia. We excluded other causes of ataxia such as other viral infections, paraneoplastic diseases, or intoxication based on medical history, laboratory results and course of the disease. There was no clinical evidence of any other autoimmune diseases related to SARS-CoV-2 infection, in particular COVID-19-associated Miller Fisher syndrome [18–21] was excluded by normal sensory functions, oculomotor function and reflexes, and COVID-19-associated opsoclonus myoclonus ataxia syndrome [12–17] and acute cerebellar ataxia with myoclonus [8–13] were excluded, since no opsoclonus or myoclonus was observed. The patient never suffered from COVID-19-related encephalopathy [22–26]. In addition, MRI scans showed no signs of cytotoxic lesions of the corpus callosum (CLOCC), which have been described recently in a fraction of patients with COVID-19-related encephalopathy [25, 26, 31]. Typical causes of sensory ataxia such as polyneuropathy or conditions with dysfunction of the posterior columns of the spinal cord were excluded clinically based on normal sensory functions and reflexes. Regarding the mild cerebellar atrophy detected in the MRI, the patient did not have any history of alcohol abuse, gluten hypersensitivity or other pre-existing cerebellar disease. However, we cannot exclude subclinical cerebellar atrophy of another, yet unidentified etiology manifested before and independent of SARS-CoV-2 infection. Interestingly, FDG-PET in 5 cases of COVD-19-associated ataxia with myoclonus and/or encephalopathy showed cerebellar hypermetabolism.

Interestingly, our patient had normal cerebellar metabolism in the FDG-PET scan as opposed to 5 cases of COVD-19-associated ataxia with myoclonus and/or encephalopathy showing cerebellar hypermetabolism [10, 23]. Since FDG-PET was performed 13 days after onset of ataxia, this may have been too late to detect abnormalities.

The mechanisms, by which SARS-CoV-2 may cause central nervous system (CNS) dysfunction, remain unclear until now, but several hypothecical explanations are currently under discussion. First, there could be direct viral neuroinvasion with neuronal damage caused by SARS-CoV-2. A case with cerebellitis associated with acute SARS-CoV-2-induced pneumonia has been described recently [7]. Interestingly, no typical MRI signs of acute cerebellitis such as cerebellar T2 hyperintensities, leptomeningeal contrast enhancement and/or cerebellar atrophy were found in this case. An example of a viral infection of the CNS, which can induce isolated cerebellar atrophy without T2 hyperintensities, is cerebellar granule cell neuronopathy caused by JC polyomavirus [32]. Such a mechanism has not been described in SARS-CoV-2 infection yet, and in our case we believe a direct SARS-CoV-2 infection of the cerebellum is highly unlikely because of normal CSF cell counts, the negative SARS-CoV-2 PCR test of the nasopharyngeal swab on day 6 of hospital admission despite persistent cerebellar symptoms, and, most importantly, because of absence of SARS-CoV-2 RNA in the CSF and intrathecal SARS-CoV-2-specific antibody production.

Another possibility described in the literature is an antiviral immune response that may manifest as coagulopathy and/or cytokine storm with cytotoxic brain lesions and/or strokes. The cytokines may cause endothelial dysfunction, activation of the coagulation cascade with microthrombi formation, vascular damage leading to hypercoagulopathy, hypoxia and microbleeds in the brain. On the other hand, cytokines that cross the blood-brain barrier may also lead to a dysregulated and excessive immune response in the brain inducing immune-mediated tissue damage [31, 33]. This mechanism is considered to induce lesions in the corpus callosum, mostly in the splenium due to high levels of cytokine and glutamate receptors in this region. However, these patients are usually clinically severely affected by encephalopathy. We did not check for inflammatory cytokines or interleukin-6 in the blood or CSF, because basic inflammatory markers in the blood such as CRP were normal and our patient was always in an acceptable general condition.

As a third and most likely explanation based on the clinical and diagnostic findings, our patient most probably suffered from a post-infectious, delayed humoral or cellular immune response cross-reactive to cerebellar autoantigens. As opposed to chronic cerebellar autoimmune diseases, post-infectious cerebellar ataxia is monophasic in most cases. Both disease entities are considered to be caused by cross-reactivity of antiviral antibodies and/or T cells to cerebellar autoantigens. Classical examples of autoimmune cerebellar ataxia are GAD antibody-associated and paraneoplastic Jo antibody-associated cerebellar ataxia. In both cases, antibodies target intracellular antigens of cerebellar neurons. An in vitro study showed extensive accumulation of anti-Jo antibodies in Purkinje cells after internalization leading to cell death and cerebellar degeneration [34]. In GAD antibody-associated disease, a direct pathogenicity of the antibody could not be proven so far, but there is evidence of T cell-mediated autoreactivity [35]. GAD-specific cytotoxic T cell infiltrates associated with neuronal damage and microglial activation were found in post-mortem histopathological studies of patients with GAD antibody-associated encephalitis [35]. Whether immune-mediated damage of cerebellar neurons or another cause before and independent of SARS-CoV-2 infection induced mild cerebellar atrophy in our case, remains open.

This case adds to the reports about SARS-CoV-2 infection-related ataxia [5–26]. Further research defining epidemiological, clinical, laboratory and therapy response characteristics is needed to prove association or causality regarding neurological dysfunctions. It is also of utmost importance to define transient and persisting, i.e. residual, deficits and organ dysfunctions after COVID-19 and we recommend to follow-up patients after COVID-19 and neurological manifestations. Our findings should encourage clinicians to critically search for less common neurological signs and symptoms during and shortly after SARS-CoV-2 infection. The latter should be considered as a potential trigger of post-infectious, probably autoimmune-mediated neurological symptoms.

## Data Availability

The datasets used and/or analysed during the current study are available from the corresponding author on reasonable request.

## Abbreviations

CMV: Cytomegalovirus
COVID-19: Coronavirus disease 2019
CSF: Cerebrospinal fluid
DNA: Desoxyribonucleic acid
FDG-PET: Fluorodeoxyglucose F 18 positron emission tomography
HSV-1: Herpes simplex virus type 1
JCV: JC polyomavirus
MRI: Magnetic resonance imaging
PCR: Polymerase chain reaction
RNA: Ribonucleic acid
SARA: Scale for the Assessment and Rating of Ataxia
SARS-CoV-2: Severe acute respiratory syndrome virus 2
VZV: Varicella zoster virus
